# Controllable synthesis of ultrathin Co_3_O_4_ and construction of RuO_2_/Co_3_O_4_ interface for oxygen evolution performance

**DOI:** 10.1039/d6ra03878f

**Published:** 2026-09-01

**Authors:** Changbao Zhao, Yuping Lin, Wen Ai, Shuyan Hua, Bin Wang, Xinle Xiao

**Affiliations:** a Anhui Engineering Research Center of Highly Reactive Micro-Nano Powders, Chizhou University Chizhou 247000 China xlxiao@czu.edu.cn; b School of Materials and Environmental Engineering, Bohai University Jinzhou 121013 China wangbinlhx@163.com

## Abstract

Replacing noble metal catalysts with transition metal oxides is a key goal in catalysis research. However, obtaining well-defined ultrathin oxides remains challenging, unlike the relatively facile preparation of typical ultrathin crystals. Here, we report the vapor–liquid–solid (VLS) growth of ultrathin Co_3_O_4_ nanostructures, using CoCl_2_ as the precursor in combination with an oxygen-containing atmosphere. Single-crystalline, micrometer-scale, thickness-tunable epitaxial Co_3_O_4_ nanosheets were successfully synthesized on Al_2_O_3_(0001) substrates and displayed favorable oxygen evolution activity following the loading of RuO_2_. During the growth process, the cobalt-containing liquid intermediate and the dynamic liquid–solid interface play crucial roles in guiding the epitaxial alignment and promoting expansion of the oxide layer. This method enables the fabrication of structurally well-defined Co_3_O_4_ with tunable surface morphology and crystallographic orientation, and offers an ideal model system for investigating structure-dependent catalytic mechanisms and surface reactivity in transition metal oxide catalysts.

## Abstract 2

Replacing noble metal catalysts with transition metal oxides is a key goal in catalysis research.

## Graphical abstract

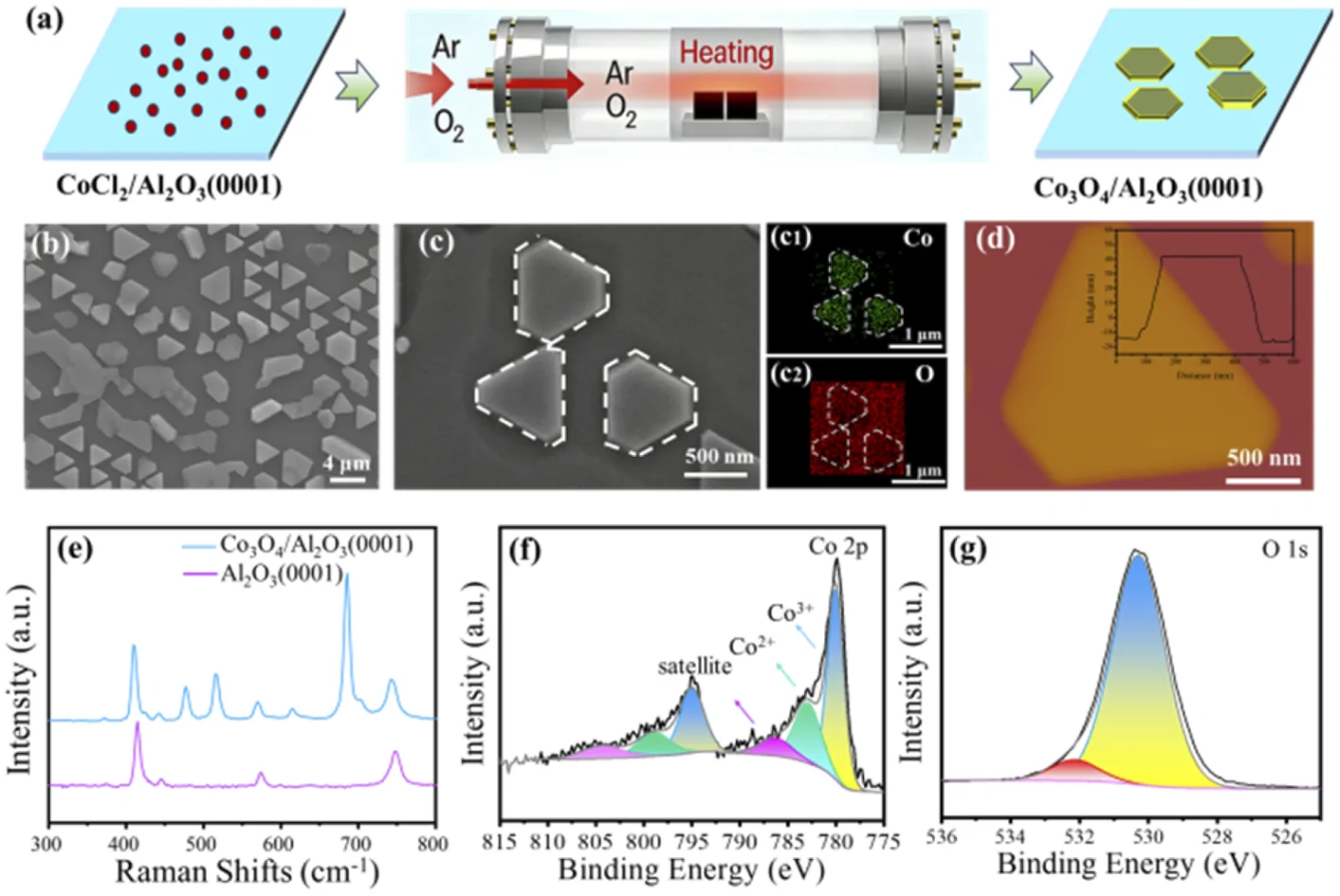

## Introduction

Transition metal oxides, characterized by their abundant reserves in the earth's crust, low cost and distinctive physicochemical properties, represent a promising class of materials for catalytic applications.^1–3^ One key objective in catalysis research is to replace or reduce the reliance on noble metal catalysts by utilizing such oxides.^4–6^ These materials can serve either as active components or supports in a wide range of catalytic processes, such as CO conversion, the oxygen evolution reaction in water electrolysis and water–gas shift reaction.^7–9^ As a representative spinel-type transition metal oxide, Co_3_O_4_ has garnered significant interest due to its outstanding catalytic performance in catalysis. This high activity stems from a suite of favorable properties, such as its relatively low Co–O bond energy, abundant reactive oxygen species, distinct cobalt ion valence variability and excellent hydrothermal stability.^10,11^ Co_3_O_4_ has been extensively studied and employed in various catalytic combustion reactions owing to its multiple valence states, abundant surface reactive oxygen species, excellent poison resistance and high thermal stability. As a typical p-type semiconductor, its variable oxidation states and the relatively low stability during redox processes facilitate substantial oxygen adsorption on the surface, thereby promoting oxygen activation and subsequent catalytic oxidation of reactants.^12^

Furthermore, it is noteworthy that the morphology of Co_3_O_4_ significantly influences its catalytic activity. For instance, Bai *et al.* synthesized Co_3_O_4_ nanoparticles, two dimensional-Co_3_O_4_ (2D-Co_3_O_4_) and three dimensional-Co_3_O_4_ (3D-Co_3_O_4_) and evaluated their performance in the catalytic oxidation of formaldehyde, respectively. The results demonstrated that 3D-Co_3_O_4_ exhibited superior activity compared to 2D-Co_3_O_4_ and nano-Co_3_O_4_.^13^ Characterization revealed that the enhanced formaldehyde oxidation performance of 3D-Co_3_O_4_ could be attributed to its three-dimensional porous channel structure, larger specific surface area, abundant reactive surface oxygen species and exposed active Co^3+^ cation sites on the (220) crystal facets. Similarly, Fan *et al.* synthesized Co_3_O_4_ in sheet, rod and cube morphologies. Among them, the rod-like Co_3_O_4_ exhibited the lowest ignition temperature for HCHO oxidation, achieving complete conversion at 120 °C with excellent stability.^14^ This superior performance was attributed to its highest specific surface area and the richest surface concentration of Co^3+^ and –OH groups.

Additionally, 2D Co_3_O_4_ as a support for metal loading has been widely used in catalytic reactions. For instance, Li *et al.* proposed an innovative stirring-assisted molten salt strategy, which successfully synthesized ultrathin 2D Co_3_O_4_ catalysts and achieved precise site-specific doping of Cu and Zn atoms.^15^ This metal doping strategy significantly enhanced the catalytic oxidation performance of toluene within a specific temperature window. Through a facile tandem configuration, the catalyst demonstrated exceptional catalytic oxidation performance for toluene across a broad temperature range. Gao *et al.* reported a nitrogen-mediated 2D cobalt oxide catalyst that demonstrates excellent oxygen evolution reaction activity and stability.^16^ The introduction of nitrogen promotes a shift in the reaction mechanism toward the lattice oxygen oxidation pathway and forms an oxygen–nitrogen–boron state acting as an electron donor. This ensures structural stability while enabling high-performance hydrogen production *via* anion exchange membrane water electrolysis. 2D cobalt oxide holds significant importance in catalysis due to its atomically thin nature and unique layered structure. It provides abundant exposed active sites, which allows optimization of reaction pathways through modulation of morphology and electronic structure, and therefore exhibits high activity and stability in energy and environment related catalytic processes such as the oxygen evolution reaction and the oxidation of volatile organic compounds.^17,18^ Although 2D Co_3_O_4_ holds considerable promise for catalytic applications, its controllable synthesis remains challenging. The conventional VSS route tends to produce irregular polycrystalline aggregates rather than well-defined single-crystalline sheets with uniform morphology, which greatly impedes the elucidation of its structure–performance correlations. Chemical vapor deposition (CVD) is widely regarded as a key method for producing high-quality ultrathin nanostructures.^19–21^ However, CoO_*x*_ prepared by the conventional CVD method does not exhibit a well-defined 2D structure.^22–24^ Notably, liquid substrates offer several advantages during material growth. With quasi-atomically smooth surfaces and high surface diffusion rates, liquid substrates can avoid the inherent defects and grain boundaries compared to solid substrates.^25^ Meanwhile, liquid precursors can form solid–liquid interfaces with the substrate, enabling controllable growth of ultrathin materials.^26,27^ Therefore, the vapor–liquid–solid (VLS) mechanism for ultrathin growth calls for further exploration.

In this work, we report the controlled synthesis of ultrathin Co_3_O_4_*via* the VLS mechanism. CoCl_2_ is used as the precursor, forming a liquid–solid interface with the sapphire substrate. Subsequent oxidation in O_2_ produces ultrathin Co_3_O_4_. The morphology and thickness of the obtained Co_3_O_4_ were characterized by scanning electron microscope (SEM) and atomic force microscope (AFM), while its chemical state and crystal structure were examined by X-ray photoelectron spectroscopy (XPS), X-ray diffraction analysis (XRD) and high-resolution transmission electron microscopy (HRTEM). By varying the growth temperature, we demonstrated that the liquid–solid interface facilitates the formation of ultrathin Co_3_O_4_. This study offers an effective strategy for fabricating model Co_3_O_4_ nanostructures and lays a foundation for further research and applications of thin oxide film materials.

## Experiments

### Substrate preparation

Al_2_O_3_(0001) substrates were cleaned through multiple cycles using ethanol and deionized water sequentially, followed by thorough rinsing with copious deionized water and drying under a nitrogen stream. To enhance surface hydrophilicity, selected substrates were subsequently subjected to the UV-ozone treatment for 30 minutes.

### Preparation of ultrathin Co_3_O_4_ sheet and RuO_2_/Co_3_O_4_

To prepare the Co_3_O_4_ nanostructure, a 0.2 mL aliquot of CoCl_2_·6H_2_O aqueous solution (200 mg mL^−1^) was spin-coated onto the Al_2_O_3_(0001) substrate at 5000 rpm. The sample was loaded into a quartz tube and placed in a thermal tube furnace. Co_3_O_4_ was deposited at 1000 °C under a flow of 100 sccm high-purity Ar as the carrier gas, with a heating rate of approximately 10 °C min^−1^. Upon reaching the target temperature, 5 sccm O_2_ was introduced into the Ar gas stream at ambient pressure. After a growth duration of 120 min, the O_2_ supply was ceased, and the system was cooled to room temperature under a continuous Ar atmosphere. The as-prepared Co_3_O_4_ was immersed in a RuCl_3_ solution, followed by drying, and then calcined at 500 °C in air to obtain two-dimensional RuO_2_/Co_3_O_4_.

### Electrochemical measurements

The oxygen evolution reaction (OER) performances were assessed in 1.0 M KOH media on an electrochemical workstation with a standard three-electrode system. A Pt net, an Hg/HgO electrode, and the as-prepared Ru/Co_3_O_4_ catalyst were employed as the counter, reference electrode and working electrode, respectively. The linear sweep voltammetry (LSV) curves were collected at a scan rate of 5 mV s^−1^. Electrochemical impedance spectroscopy (EIS) was investigated with amplitude of 5 mV and the frequency range from 10 kHz to 0.01 Hz. The cyclic voltammetry (CV) curves with different scan rates were obtained under non-faradaic region.

## Results and discussion

**Fig. 1:**
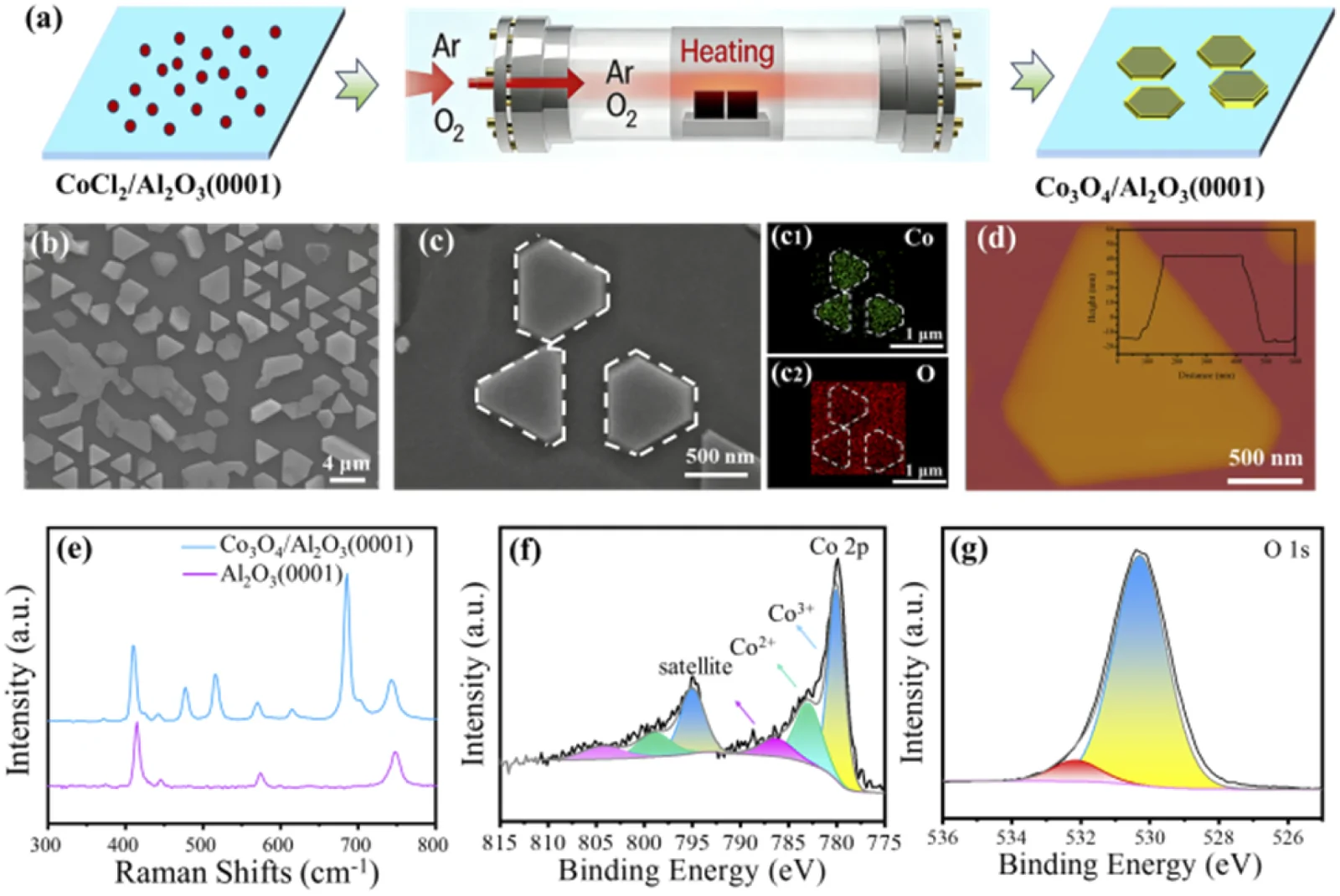
(a) Schematic illustration of the VLS growth process of Co_3_O_4_. (b) and (c) Typical SEM images of the CoO_*x*_ nanosheets and energy dispersive X-ray (EDX) elemental mappings of Co and O of the CoO_*x*_ nanosheets. (d) Typical atomic force microscopy (AFM) image of the CoO*x* nanosheets; (e) Raman spectra of the CoO_*x*_ nanosheets; (f) and (g) corresponding high-resolution XPS Co 2p and O 1s spectra.

Based on the VLS growth mechanism, ultrathin CoO_*x*_ overlayers were synthesized on Al_2_O_3_(0001) substrates *via* an ambient-pressure CVD route using CoCl_2_ as the precursor (Fig. 1a). Initially, a uniform precursor layer was deposited by spin-coating the substrate with an aqueous solution of CoCl_2_·6H_2_O (*e.g.*, 200 mg mL^−1^) followed by drying in air. The coated substrate was then placed into a quartz tube furnace and heated to 1000 °C under a 100 sccm Ar flow with a heating rate of 10 °C min^−1^. Upon reaching the target temperature, 5 sccm O_2_ was introduced into the gas stream to initiate oxidation and crystallization. After a dwell time of 120 min, the O_2_ supply was shut off and the system was cooled to room temperature under pure Ar, resulting in the formation of triangular and hexagonal ultrathin CoO_*x*_ on the Al_2_O_3_(0001) surface.

As shown in Fig. 1b, the SEM image displays that the Al_2_O_3_(0001) surface exhibits a uniformly deposited nano crystals, achieving a high surface coverage of approximately 60%. Fig. 1c reveals that the flake on the Al_2_O_3_(0001) surface predominantly exhibits triangular or hexagonal structures. The lateral dimensions of the triangular and hexagonal flake structures predominantly fall within the range of 500 nm to 2 µm. The EDX mapping image (Fig. 1c1) reveals a homogeneous distribution of cobalt (Co) throughout the nanosheets. Due to the intrinsically strong oxygen (O) signal originating from the Al_2_O_3_(0001) substrate, the O element also exhibits a uniform distribution over the selected region (Fig. 1c2). These results collectively demonstrate that the nanosheets are composed of cobalt and oxygen, and that these ultrathin nanostructures possess excellent compositional uniformity.

The morphology and thickness of individual CoO_*x*_ nanosheet were analyzed by atomic force microscopy (AFM) (Fig. 1e). The AFM image reveals that the CoO_*x*_ nanosheet possess a relatively smooth surface, and the thickness is approximately 60 nm as indicated in the inset of Fig. 1e. Raman spectroscopy was conducted to analyze the composition and structure of the nanosheets grown on the Al_2_O_3_(0001) surface (Fig. 1e). The Raman spectrum displays the characteristic signatures at approximately 482, 522, 620, and 690 cm^−1^ which belong to the Co_3_O_4_ spinel phase. These characteristic signatures are ascribed to the E_g_, F2g^2^, F2g^3^ and A_1g_ vibrational modes, respectively. The excellent agreement of these peak positions with reference data confirms the formation of a well-crystallized, phase-pure Co_3_O_4_ material.^28^

XPS spectra were acquired to analyze the elemental composition and valence states of the pure Co_3_O_4_. The high-resolution Co 2p XPS spectrum of Co_3_O_4_ displays two broad peaks centered at approximately 781 and 796 eV, separated by a spin–orbit splitting of about 15 eV, characteristic of the Co 2p_3/2_ and Co 2p_1/2_ levels. This spectral feature confirms the presence of Co_3_O_4_ in the sample. Deconvolution of the spectrum further reveals that the peaks at 780.9 and 796.5 eV correspond to Co^3+^ 2p_3/2_ and Co^3+^ 2p_1/2_, respectively, while those at 782.6 and 798.3 eV are assigned to Co^2+^ 2p_3/2_ and Co^2+^ 2p_3/2_. Additionally, two satellite features are observed at 786.9 and 803.6 eV, associated with the Co^2+^ and Co^3+^ species, respectively (Fig. 1f). The O 1s XPS spectra are deconvoluted into two peaks (Fig. 1g), corresponding to lattice oxygen (530.3 eV) and surface-adsorbed species (532.3 eV), the latter comprising contributions from carbonate and water.^29,30^

**Fig. 2:**
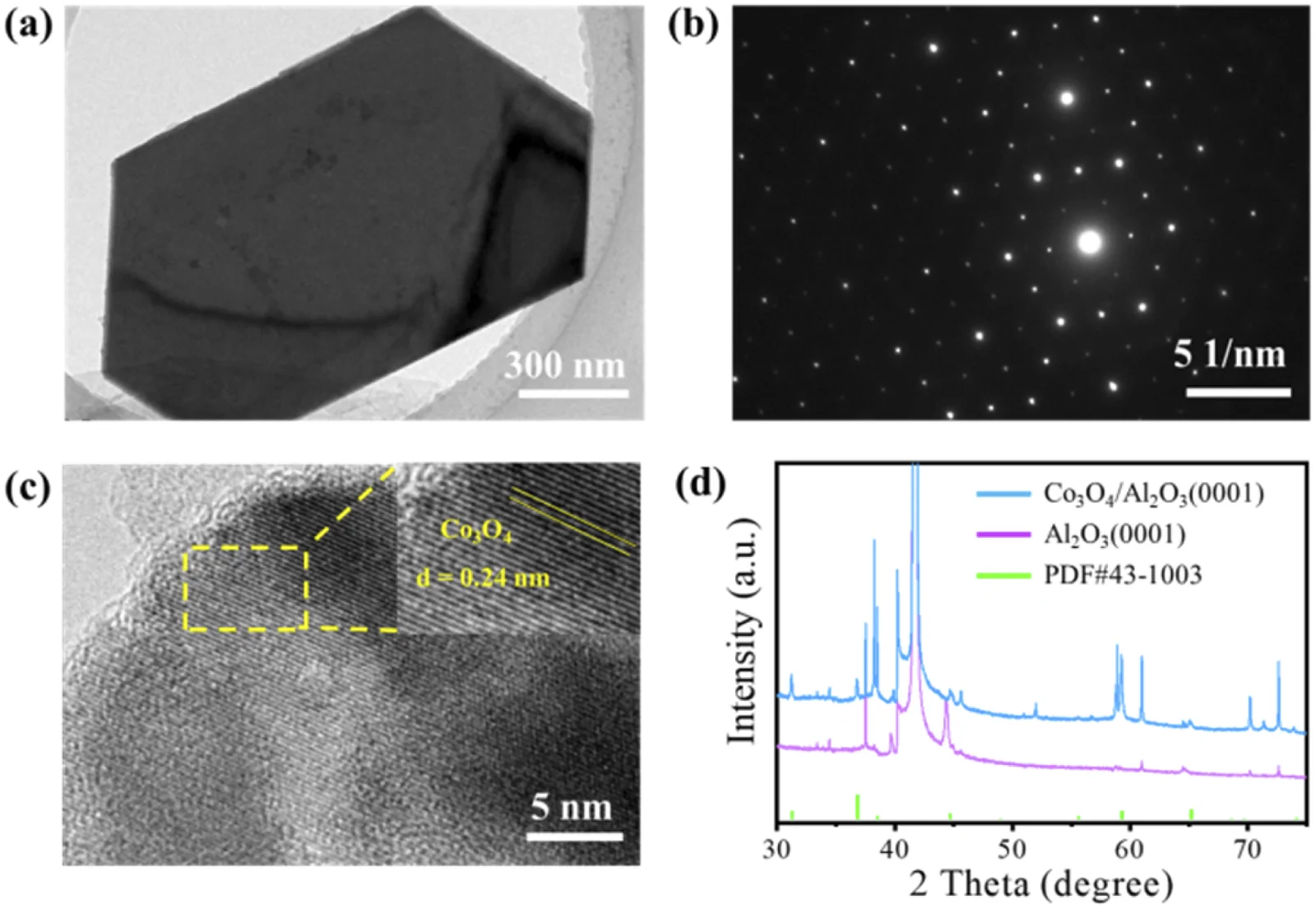
(a) Typical transmission electron microscopy (TEM) images of CoO_*x*_ nanosheets; (b) corresponding selected area electron diffraction pattern of CoO_*x*_ nanosheets; (c) high-resolution TEM (HRTEM) image of CoO_*x*_ nanosheets; (d) X-ray diffraction (XRD) pattern of the Co_3_O_4_/Al_2_O_3_(0001) surface.

The typical TEM images presented in Fig. 2a provide direct evidence that the material primarily consists of hexagonal nanostructures. These images reveal a sheet-like assembly with lateral dimensions extending up to several hundred nanometers. This assignment is corroborated by the SAED pattern in Fig. 2b. The sharp and discrete diffraction spots arranged in a regular pattern unambiguously demonstrate the single-crystalline character of individual Co_3_O_4_ nanosheet.^29^ The well-resolved lattice fringes in Fig. 2c attest to the high crystallinity of the nanosheets. A measured interplanar spacing of 0.24 nm provides a key identifier for the specific crystal phase.^31^ The crystalline phase was unambiguously identified by X-ray diffraction (XRD). Specifically, Fig. 2d shows that the characteristic diffraction peaks observed at 2*θ* = 31.3°, 36.8°, 38.5°, and 59.3° correspond to the (220), (311), (222), and (511) planes of the Co_3_O_4_ phase, respectively.^30^ These well-defined peaks match the reference and confirm the successful formation of the Co_3_O_4_ phase. Nevertheless, additional characteristic peaks present at 2*θ* = 38.2° and 72.6° are assigned to the (015) and (027) crystal planes of the unreacted CoCl_2_ (PDF#15-0381), respectively. Overall, the integrated data from TEM, HRTEM, SAED, and XRD collectively demonstrate that the synthesized product consists of polycrystalline, ultrathin Co_3_O_4_ nanosheets with a well-defined crystal structure and high phase purity. The measured interplanar spacing of 0.24 nm, the single-crystal diffraction pattern and the matching XRD reflections together furnish robust confirmation of its crystallographic identity.

Currently, the typical synthesis of CoO_*x*_ predominantly follows a vapor–solid–solid (VSS) pathway, wherein CoCl_2_ is heated in an oxygen-containing atmosphere.^32,33^ During this process, the oxidation of CoCl_2_ to CoO_*x*_ occurs at elevated temperatures (∼500 °C) in the presence of air and moisture. The transformation is primarily driven by a hydrolysis-oxidation mechanism that CoCl_2_ first hydrolyzes to form intermediate species such as Co(OH)Cl or Co(OH)_2_, releasing HCl.^34^ These intermediates are subsequently oxidized by O_2_, yielding CoO_*x*_ as the final product. To further demonstrate the advantages of the VLS mechanism in preparing 2D CoO_*x*_ sheets, we synthesized CoO_*x*_ by heating CoCl_2_ as a precursor in O_2_ at 700 °C.

**Fig. 3:**
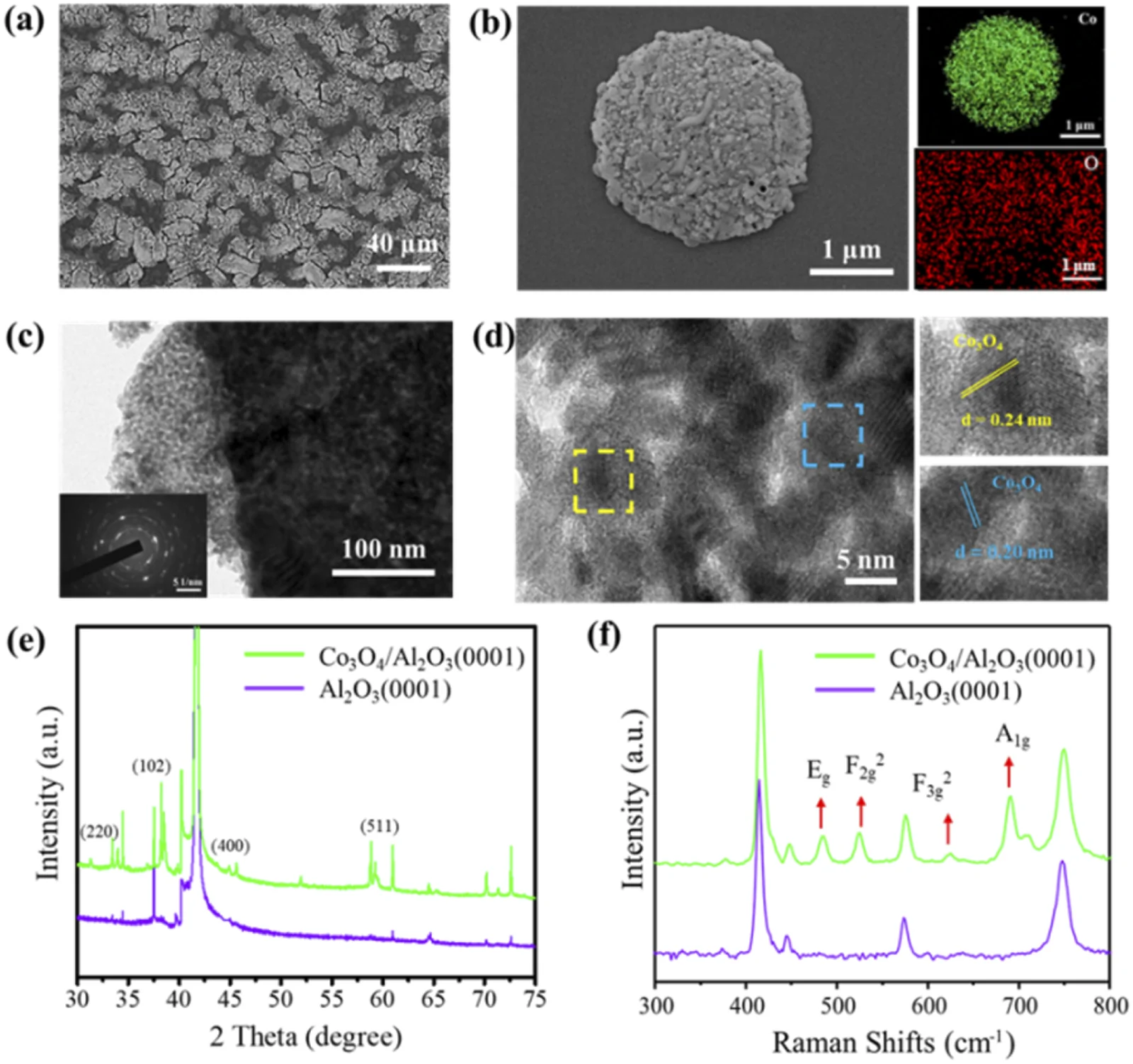
(a) Typical SEM images of the CoO_*x*_ powder *via* VSS growth process. (b) EDX elemental mappings of Co and O of the CoO_*x*_ powder; (c) typical TEM images of the CoO_*x*_ powder *via* VSS growth process; (d) HRTEM image of CoO_*x*_ powder. (e) XRD pattern of the CoO_*x*_ powder; (f) Raman spectra of the CoO_*x*_ powder.

As shown in Fig. 3a, the SEM image reveals that the CoO_*x*_ powder synthesized *via* the VSS growth mechanism exhibit a distinct and irregular morphology. Unlike highly ordered nanosheets, the obtained material consists of aggregated, sheet-like domains with non-uniform lateral dimensions and poorly defined edges. The surfaces of these domains are notably rough, displaying a granular or textured appearance at higher magnification. This observed irregular morphology and surface smoothness is characteristic of the VSS growth process under the employed conditions, where heterogeneous nucleation and non-equilibrium kinetics predominate. The resulting irregular and rough morphology can be attributed to the limited surface diffusion of adatoms inherent to this mechanism, which ultimately influences the materials specific surface area and potential surface reactivity. Elemental mapping analysis *via* EDX nevertheless reveals that the irregular morphology is uniformly composed of cobalt and oxygen, with both elements being homogeneously distributed throughout the structure (Fig. 3b). As shown in Fig. 3c, TEM analysis suggests that the material primarily consists of polycrystalline domains, where grain aggregation and domain boundaries are clearly visible. And the selected-area electron diffraction SAED pattern in Fig. 3c displays continuous polycrystalline diffraction rings, further confirming its polycrystalline structure. The HRTEM image in Fig. 3d reveals interplanar spacings of 0.24 nm and 0.20 nm, corresponding respectively to the (311) and (400) planes of the spinel Co_3_O_4_ phase.^35^

To establish the nature of the crystalline phase, the Co_3_O_4_/Al_2_O_3_(0001) powder sample was characterized by XRD. The XRD pattern exhibits characteristic diffraction peaks at 2*θ* ≈ 31.3°, 38.5°, 44.8°, and 59.3°, which match well with the standard reference for Co_3_O_4_ (PDF#42-1467). These peaks are indexed to the (220), (222), (400), and (511) planes, respectively. The sharp peak profiles and the absence of impurity signals demonstrate that the as-prepared material is a well crystallized, phase-pure Co_3_O_4_ (Fig. 3e). As shown in Fig. 3f, Raman spectroscopy confirms the formation of high-purity Co_3_O_4_ with a well-defined spinel structure. The sharp and distinct peaks observed at ∼480 (E_g_), 520 (F_2g_), 620 (F_2g_), and 690 cm^−1^ (A_1g_) indicate good crystallinity and structural order. The absence of extra modes further verifies that no significant secondary phases or structural defects are present in the sample. Taken together, these results demonstrate that the Co_3_O_4_ produced by the VSS growth mechanism under non-equilibrium conditions lacks both a 2D sheet-like structure and a well-defined surface morphology, displaying instead an overall irregular and rough morphology.

**Fig. 4:**
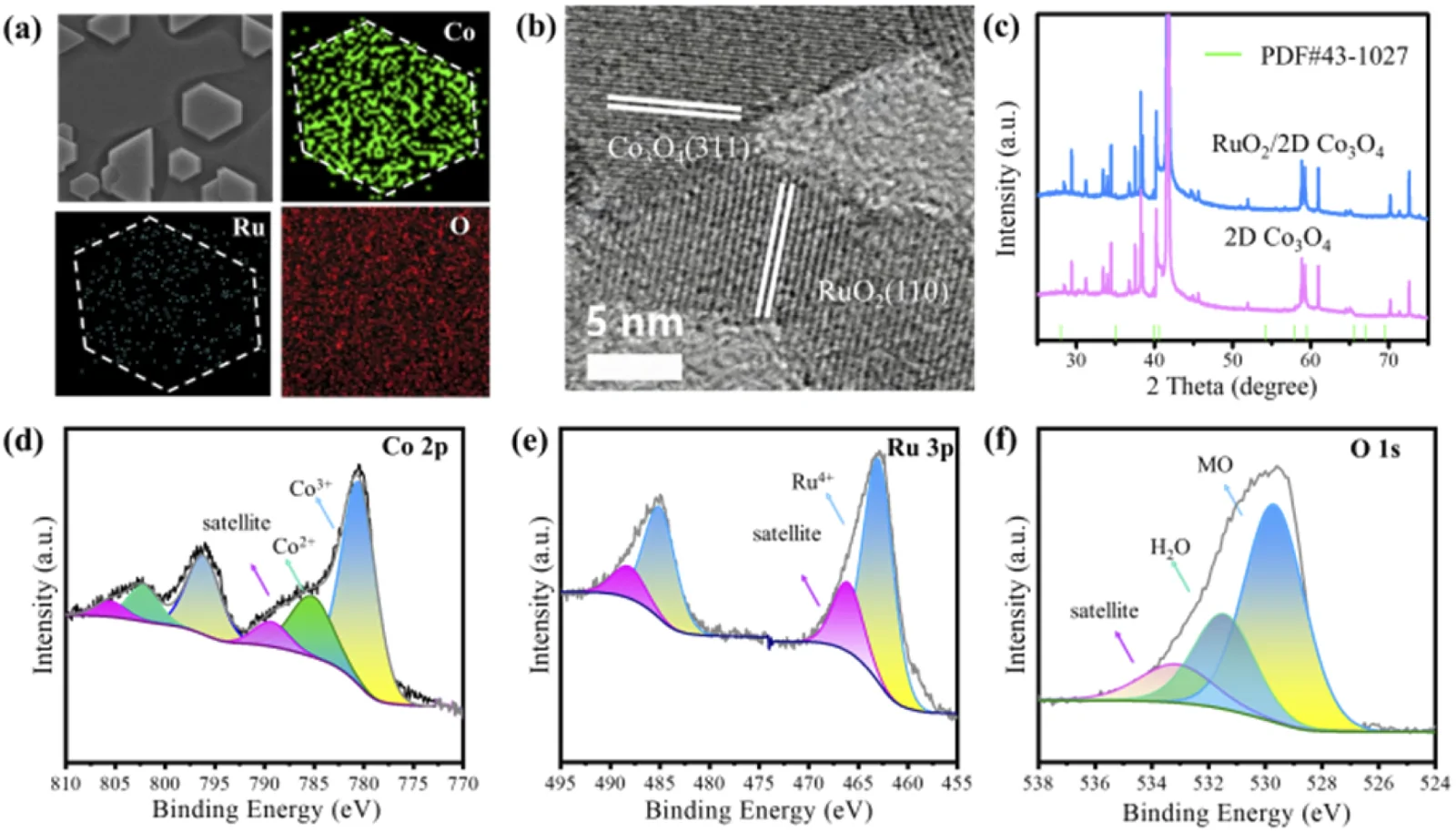
(a) SEM image and elemental EDX mapping of the 2D RuO_2_/Co_3_O_4_ structure; (b) TEM image of the 2D RuO_2_/Co_3_O_4_ structure; (c) XRD pattern of the 2D RuO_2_/Co_3_O_4_ structure; (d)–(f) corresponding high-resolution XPS Co 2p, Ru 3p, and O 1s spectra.

Following confirmation of the successful preparation of ultrathin epitaxial 2D Co_3_O_4_, we aimed to further enhance its OER performance. Although RuO_2_ possesses high intrinsic OER activity, it suffers from insufficient durability. Constructing a heterointerface between RuO_2_ and Co_3_O_4_ enables interfacial electronic modulation, yielding a highly active catalyst, while Co_3_O_4_ contributes structural stability. The synergy between the two components optimizes the adsorption energetics of oxygenated intermediates. Accordingly, we selected the ultrathin epitaxial Co_3_O_4_ as the support to combine its superior electronic conductivity with the high activity of RuO_2_, fabricating a 2D RuO_2_/Co_3_O_4_ composite for systematic OER evaluation. The 2D Co_3_O_4_ was immersed in a RuCl_3_ solution of a given concentration, followed by drying and calcination at 400 °C. Inductively coupled plasma (ICP) analysis revealed that the resulting 2D RuO_2_/Co_3_O_4_ composite catalyst possessed a Ru loading of 1.38 wt%. As shown in Fig. 4a, the SEM and EDX mapping results reveal that the surface of the 2D Co_3_O_4_ retains a well-defined morphology, with Co, Ru, and O elements uniformly distributed throughout the 2D structure. HRTEM results reveal the presence of the (110) plane of RuO_2_ and the (311) plane of Co_3_O_4_, indicating the formation of a well-defined interface between them (Fig. 4b). The XRD results confirm that the 2D RuO_2_/Co_3_O_4_ sample consists of two phases, RuO_2_ (JCPDS no. 40-1290) and Co_3_O_4_ (JCPDS no. 43-1003). The diffraction peaks of the Co_3_O_4_ phase correspond well to those of 2D Co_3_O_4_, while the signals from RuO_2_ are relatively weak due to its low content (Fig. 4c). The chemical composition and elemental states of the 2D RuO_2_/Co_3_O_4_ were further analyzed by XPS spectroscopy. The Co 2p peaks can be deconvoluted into six components, which are assigned to Co^2+^, Co^3+^, and carbonate species (Fig. 4d). Compared with pristine Co_3_O_4_, the Co 2p binding energy of the composite shifts to lower energy by 0.3 eV. In contrast, as shown in Fig. 4e, the Ru 3p binding energy shifts to higher energy after compositing. These results suggest a strong interaction between RuO_2_ and Co_3_O_4_ in the 2D RuO_2_/Co_3_O_4_, manifested as electron transfer from RuO_2_ to Co_3_O_4_. The O 1s spectrum provides additional evidence for the chemical states of Co and Ru (Fig. 4f).

**Fig. 5:**
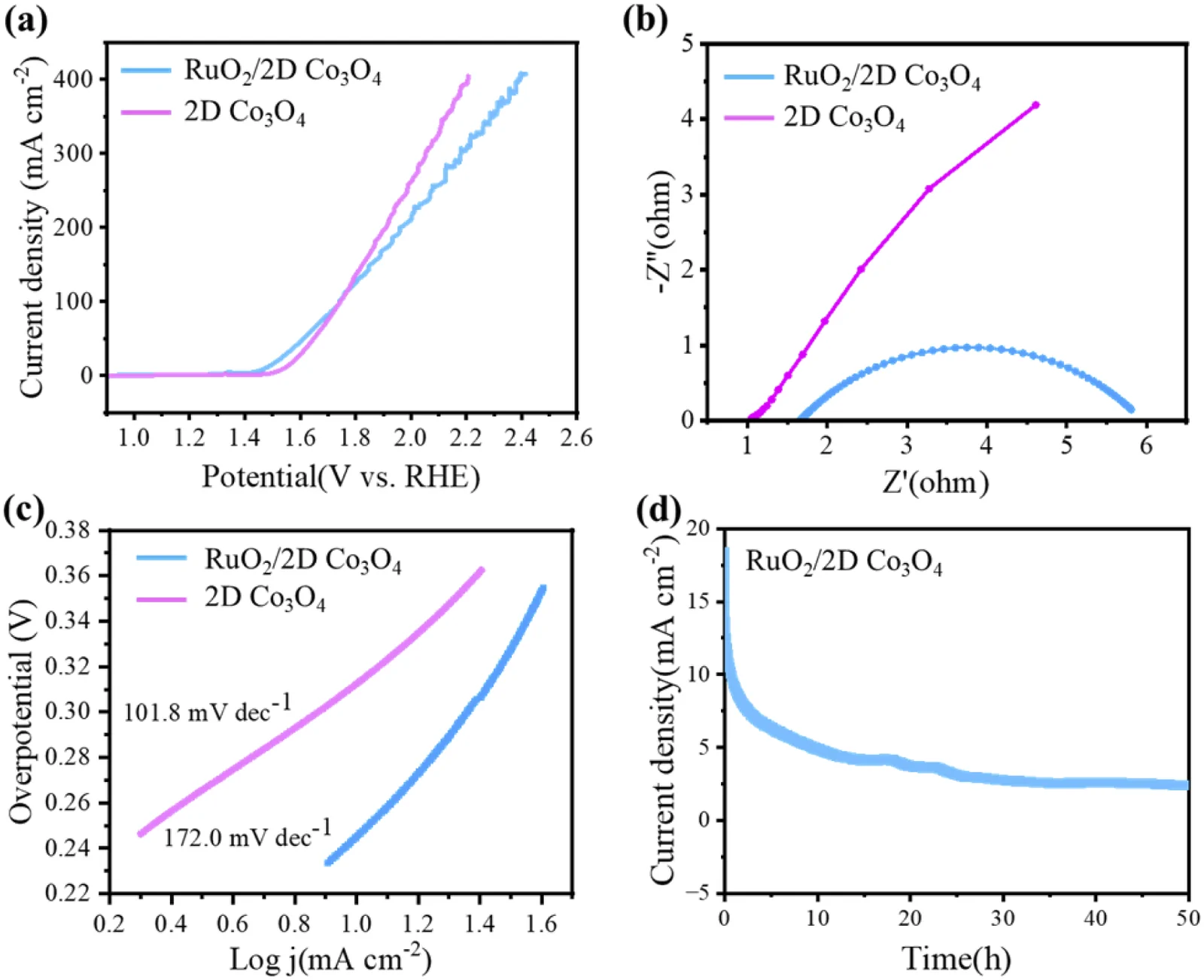
The electrochemical properties of the RuO_2_/2D Co_3_O_4_ and pure 2D Co_3_O_4_ tested in 1 M KOH electrolyte. (a) LSV polarization curves; (b) Nyquist plots; (c) Tafel slopes; (d) chronopotentiometry curve of the RuO_2_/2D Co_3_O_4_.

The electrochemical performance tests were conducted on the 2D RuO_2_/Co_3_O_4_ catalyst. The oxygen evolution reaction (OER) performance of the catalyst was evaluated in 1.0 M KOH electrolyte. Linear sweep voltammetry (LSV) measurements reveal that the RuO_2_/2D Co_3_O_4_ catalyst achieves a current density of 10 mA cm^−2^ at an overpotential of only 244 mV, whereas the pristine 2D Co_3_O_4_ requires an overpotential of 312 mV to reach the same current density. This distinct comparison clearly demonstrates that the interfacial structure formed between RuO_2_ and the ultrathin Co_3_O_4_ significantly enhances the intrinsic OER activity (Fig. 5a). As a result, the overall catalytic performance of the hybrid is superior to that of most transition metal oxide-based OER catalysts reported in the literature.^36–39^ Electrochemical impedance spectroscopy (EIS) measurements reveal that the RuO_2_/2D Co_3_O_4_ composite exhibits a charge transfer resistance of 4.29 Ω, which is substantially lower than that of bare Co_3_O_4_ (Fig. 5b). Such a low interfacial charge transport resistance suggests that the incorporation of RuO_2_ effectively enhances the reaction kinetics at the catalyst–electrolyte interface, substantially improving charge transfer efficiency.^40,41^ The Tafel slope of RuO_2_/2D Co_3_O_4_ (172 mV dec^−1^) is larger than that of pristine 2D Co_3_O_4_ (101.8 mV dec^−1^), indicating a stronger potential dependence of the OER kinetics after hybridization, while the significantly lower overpotential confirms the beneficial interfacial effect (Fig. 5c). The RuO_2_/2D Co_3_O_4_ catalyst exhibits outstanding long-term durability toward the OER in alkaline media, with negligible activity degradation after prolonged electrolysis (Fig. 5d). Overall, the RuO_2_/Co_3_O_4_ heterointerface design synergistically boosts both activity and stability. This is attributed to interfacial charge redistribution, which optimizes the adsorption energetics of intermediates, alongside strong chemical anchoring that effectively inhibits Ru dissolution. Concurrently, the engagement of the lattice-oxygen participation pathway lowers the reaction energy barrier, resulting in enhanced catalytic turnover.

**Scheme 1:**
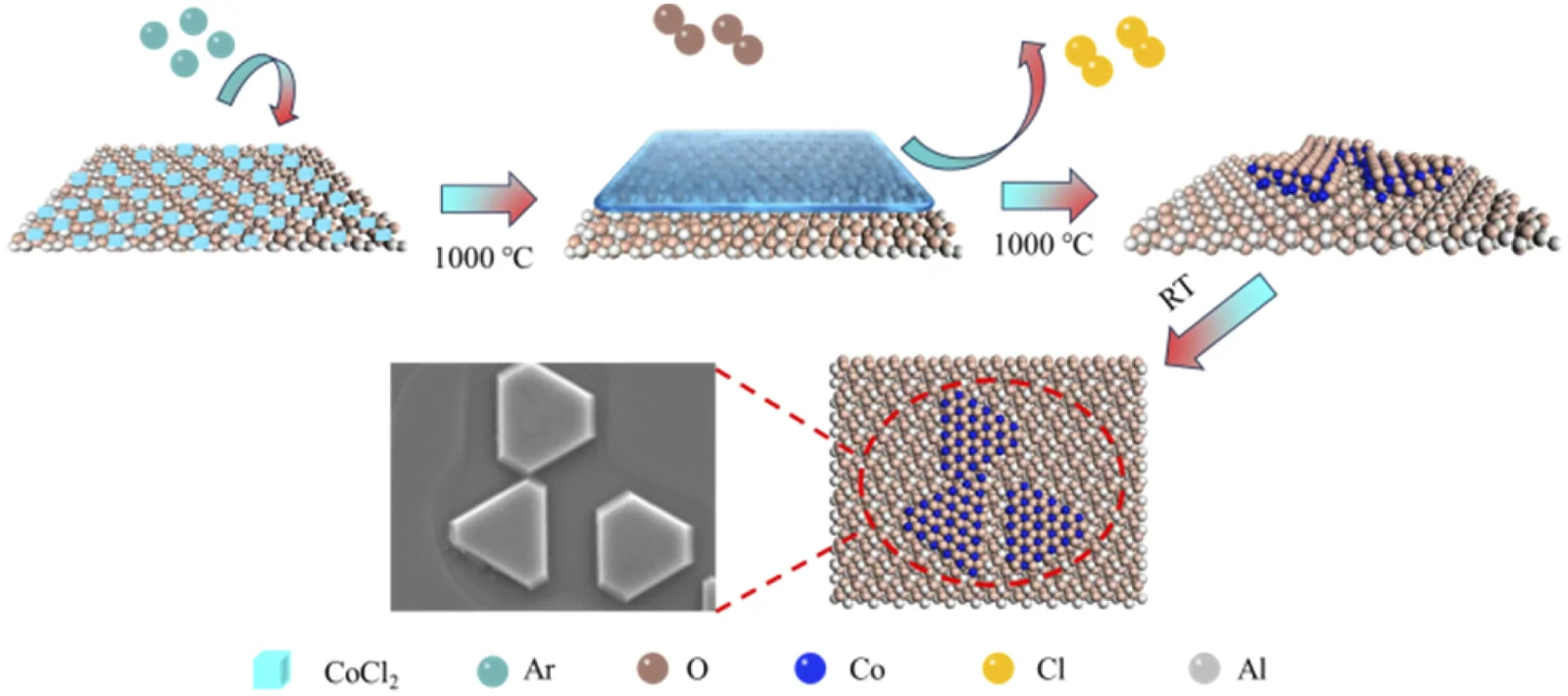
Schematic illustration of the growth of ultrathin Co_3_O_4_ nanosheets.

In this work, high-temperature liquid CoCl_2_ is used as a precursor for the epitaxial growth of ultrathin Co_3_O_4_ on Al_2_O_3_(0001) substrates. The proposed growth mechanism is depicted in Scheme 1. We suggest that the key driving force is a liquid-film-confined chemical reaction, which, in combination with precise lattice matching at the heterointerface, likely leads to a cooperative and oriented structural transformation. The process begins with the melting and spreading of the precursor. Under heating at approximately 1000 °C, solid CoCl_2_ melts into a liquid phase and uniformly spreads to form a continuous liquid film due to its moderate wettability on the Al_2_O_3_(0001) surface. This liquid film undergoes a series of sequential chemical and structural transformations in an introduced O_2_ atmosphere. First, surface oxidation and hydrolysis reactions occur at the gas–liquid interface, where Co^2+^ reacts with adsorbed oxygen and trace amounts of water vapor from the environment, gradually constructing a Co–O–Co network.^42^ Concurrently, Cl^−^ is considered to be liberated as volatile Cl_2_, which is likely to lower the system's free energy considerably, thereby imparting an irreversible thermodynamic impetus to the reaction. Thereafter, at the liquid-film/substrate interface, the amorphous Co–O intermediate seems to be directed toward epitaxial crystallization under the structural templating influence of the underlying substrate lattice. *Via* interfacial atomic rearrangement and ordering, the intermediate is anticipated to convert orientationally into spinel-structured Co_3_O_4_ nuclei.^43^ The overall driving force for this transformation primarily stems from the entropy increase due to the volatilization of chlorine and the reduction in interfacial energy resulting from the formation of the epitaxial interface.

The maintenance of ultrathin morphology in the resulting film is attributed to the synergistic interplay of the following three key factors.^44^ First, the spreading thickness of molten CoCl_2_ is thermodynamically self-limited under specific temperature conditions and substrate surface tension.^45^ This pre-formed liquid layer physically confines the vertical growth dimension of the subsequent solid product, providing the initial geometric constraint for achieving ultrathin characteristics. Second, the Al_2_O_3_(0001) surface exhibits a hexagonal close-packed arrangement of oxygen atoms, which closely matches the oxygen sub-lattice arrangement on the (111) plane of Co_3_O_4_ crystals.^46,47^ This excellent lattice matching allows the substrate to act as an effective atomic-scale template, which guides the Co_3_O_4_ nuclei to expand laterally in a layer-by-layer epitaxial mode and strongly suppresses the tendency for 3D island growth. The oxidation reaction is simultaneously triggered at both the liquid-film–gas and liquid-film–solid (the Al_2_O_3_ substrate) interfaces and proceeds primarily along the 2D direction parallel to the substrate. The interfacial reaction proceeds at a significantly higher rate than the longitudinal diffusion of Co ions within the bulk liquid film or the solid product. The interfacial reaction rate appears to be significantly higher than the vertical diffusion rate of Co ions within the bulk liquid film or the solid product. This kinetic anisotropy may effectively confine the material transformation within the 2D plane, thereby facilitating the formation of a large-area, atomically flat, and uniformly thick continuous film.

## Conclusions

In this work, a VLS strategy based on a molten salt precursor was developed for the controlled synthesis of ultrathin Co_3_O_4_ nanostructures. Well-defined triangular and hexagonal Co_3_O_4_ nanosheets with excellent crystallinity were successfully fabricated on Al_2_O_3_(0001) substrates. Mechanistic investigations reveal that the favorable wettability of molten CoCl_2_ at high temperatures and the formation of a dynamic liquid–solid interface with the substrate are critical for achieving 2D lateral confined growth. Compared with the conventional VSS route, the VLS mechanism effectively prevents polycrystalline aggregation, yielding structurally well-defined single-crystalline materials. Furthermore, we successfully constructed a 2D RuO_2_/Co_3_O_4_ interfacial structure, which served as a catalyst for the OER and exhibited excellent catalytic activity. This work provides a new strategy for the controlled synthesis of ultrathin oxide materials and holds significant implications for the fields of catalysis and surface science.

## Author contributions

Bin Wang and Xinle Xiao designed the experiment and analyzed the data. Changbao Zhao conducted the VLS growth and analyzed the data. Yuping Lin conducted the electrochemical measurements and analyzed the data. All the authors discussed and commented on the manuscript.

## Conflicts of interest

The authors declare that they have no known competing financial interests or personal relationships that could have appeared to influence the work reported in this paper.

## Data Availability

The data supporting this article have been included in the manuscript.
